# Fiber Bragg Gratings Sensor Strain–Optic Behavior with Different Polymeric Coatings Subjected to Transverse Strain

**DOI:** 10.3390/polym16091223

**Published:** 2024-04-27

**Authors:** Manuel González-Gallego, Félix Terroba Ramírez, Juan Luis Martínez-Vicente, Miguel González del Val, Juan José López-Cela, Malte Frövel

**Affiliations:** 1 National Institute of Aerospace Technology, ICTS–CEHIPAR, 28048 Madrid, Spain; 2 Instituto de Investigaciones Energéticas y Aplicaciones Industriales, Escuela Técnica Superior de Ingeniería Industrial, Universidad de Castilla—La Mancha, 13071 Ciudad Real, Spain; 3 National Institute of Aerospace Technology, 28850 Torrejón de Ardoz, Spain

**Keywords:** biaxial testing, polymeric coating, FBGS, composite material, transverse strain, structural health monitoring

## Abstract

This research work is based on a previous study by the authors that characterized the behavior of FBG sensors with a polyimide coating in a structural monitoring system. Sensors applied to structural health monitoring are affected by the presence of simultaneous multidirectional strains. The previous study observed the influence of the transverse strain ($\varepsilon_{y}$) while keeping the longitudinal strain constant ($\varepsilon_{x}$), where the *x* direction is the direction of the optical fiber. The present study develops an experimental methodology consisting of a biaxial test plan on cruciform specimens with three embedded FBG sensors coated with polyimide, acrylate, and ORMOCER^®^. Applying the Strain–Optic Theory as a reference, a comparison of the experimental values obtained with the different coatings was studied. This experimental work made it possible to study the influence of the transverse strain ($\varepsilon_{y}$) on the longitudinal measurements of each FBGS and the influence of the coating material. Finally, the calibration procedure was defined as well as K (strain sensitivity factor) for each sensor.

## 1. Introduction

Composite materials are an alternative to metal materials in structural construction in multiple areas [1]. In the aerospace industry, composite materials are primarily used for constructing aerostructures, particularly aircraft. They exceed 50% of the structural weight of modern aircraft [2,3,4,5]. In other industries, such as naval [6,7,8] and transport [9,10,11,12,13], the use of composite materials is more limited due to factors such as the recycling of materials fabricated from thermoset resins [14,15] and the absence of high-speed manufacturing processes. However, composite materials are trending upward. Composite materials are generally linked to the design of lightweight and optimized structures, a key factor in the aerospace sector and important to other sectors. Applied design philosophies have evolved over the last few decades, moving from structures designed for safe living to those designed for certain failure or withstanding damage, the latter philosophy being applied if a structurally optimized component is desired. The philosophy of tolerance for harm is based on two key pillars:

- Know and quantify the properties of the material, such as maximum allowable defect size or speed and propagation of the defect, among others. Both fields have been extensively studied in the specific case of composite materials [16,17,18].
- Have an inspection and maintenance policy that can detect faults before they reach critical size [19,20].

Normally, maintenance policies are based on a schedule of inspections and preventative actions. However, current trends in sectors such as aerospace, wind, or railways tend to replace this with maintenance on condition, which reduces costs significantly. One of the keys to maintenance on condition [21] involves monitoring the equipment to be maintained, which indicates their real situation at all times and when to act specifically. Structural monitoring is known as SHM (structural health monitoring) [22,23]. Its objectives are to detect stress levels in the structure, the likelihood of failure, and the depletion of its life due to fatigue or the appearance of overloads, among others [24,25,26]. One of the most widely used monitoring technologies is fiber optic sensors in Bragg gratings (FBGSs), which offer several interesting advantages over extensometry technologies, including small dimensions, the ability to be embedded, simplicity in cabling due to their multiplexability, stable thermal and load monitoring, and insensitivity to electromagnetic interference, among others [27,28,29,30,31]. Using FBGSs in structures necessitates a comprehensive understanding of the sensor’s behavior and its response to various variables, including temperature, humidity, dynamic loads, and the occurrence of loads and/or strains in multiple directions. This last aspect has been studied by multiple authors and was also the subject of an experimental study by the authors of this article [32,33,34,35,36]. Having an inspection and maintenance policy that detects faults before they reach critical size is necessary. Typically, the most commonly used coatings are polyimide, acrylate, and ORMOCER^®^ [37,38,39,40]. The importance and influence of the coating on an FBG sensor’s response lies in the very function it performs, which is to protect the core and coating from exposure to moisture and abrasion on its surface, prevent the appearance of micro-cracks and premature damage to the optical fiber, as well as guarantee the phenomenon of reflection according to the Snell Theory. According to Nath et al. [41], polyimide coatings have the advantage of being resistant to elevated temperatures up to 300 °C and providing reliable results when embedded. Polyimide, however, is sensitive to humidity. Acrylate coatings best protect fiber optics and are immune to humidity; however, temperatures from 100 °C can be critical [41]. The first acrylate coatings consisted of one layer, but due to attenuation problems induced by micro-curvatures or shear retardation, they became two layers. Gloge [42] elaborated on this study by stating that micro-curvature losses are minimized using inner (primary) and outer (secondary) coatings with an elastic modulus ratio ten times higher than the secondary versus the primary. In recent years, an ORMOCER^®^ coating formed from a combination of ceramic and metal has been used. It has a high elastic modulus, is not affected by humidity, provides better radiation protection, and is stable at temperatures above 200 °C [43,44,45].

There are different studies on coating type influence on embedded FBGSs. For example, Pak et al. [46] and Sirkis et al. [47] observed that a coating’s thickness and shear modulus influence shear-induced strain in the sensor. Roberts et al. [48] noted that using brittle materials as a coating leads to crack formation at low load levels. Other researchers have studied how bonding between the sensor and the host material influences the type of coating material and bonding agent for improving adhesion [41,49]. Recent studies on coatings have focused on temperature measurement with FBGSs. Mishra et al. [50] conducted experiments with different coatings to study how temperature sensitivity varies from the thermal expansion coefficient. Studies comparing coatings are noteworthy for the results obtained at cryogenic temperatures. Sampath et al. [51] compared composite materials’ coatings under cryogenic conditions to measure temperature and strains with and without a coating. They found that coated sensors had a sensitivity of 48 pm/°C, ten times higher than uncoated FBGSs. Metallic coatings such as gold and silver have demonstrated a marked improvement in sensor sensitivity to high-temperature gradients, with a delay response of 300 ms compared to bare sensors [52]. It is also worth mentioning that Weisbrich et al.’s study [53] on shrinkage tests, which analyzed the influence of output signals on distributed FBGSs (Rayleigh type) in concrete structures, used the same coatings studied in the present work. Their results showed that the ORMOCER^®^ coating had the least strain losses (<2%), followed by the polyimide and acrylate coatings (<4%).

The need to study the influence of the transverse strain on the FBGS response is justified by some researchers in the scientific community, such as R. M. Measures [54] and Luyckx et al. [55]. This work investigates the generation of multiaxial strain states in a cruciform specimen made of carbon-fiber-reinforced composite material under different load cases. Three FBG sensors located in the central area of the specimen were embedded with three types of coating material: polyimide, acrylate, and ORMOCER^®^. In a previous study, we examined the impact of transverse strain on longitudinal strain measurement for embedded polyimide-coated FBGSs. In this work, a non-negligible measurement error was observed in tests caused by transverse strains transmitted to the sensor, and K (strain sensitivity factor) was calculated by uniaxial characterization [36]. In the present work, a campaign of similar biaxial tests was performed by maintaining a constant longitudinal strain and varying the transverse strain using a strain gauge rosette installed in the central area to gauge measurement. The tests consisted of four cases of longitudinal strain (500 με, 1000 με, 1500 με, and 2000 με). We simultaneously varied the transverse strain between 0 με and 4000 με in steps of 500 με while keeping the longitudinal strain constant and pausing each step to stabilize the sensor. We applied the equations of Kim et al. [56], corresponding to the Strain–Optic Theory, to an isotropic sensor kept at a constant temperature. These strain values correspond to those common in composite structures for naval, aeronautics, and space use. In addition to calculating the influence of transverse strain on the sensor response by analyzing $∆\lambda_{B}$ (variation in the Bragg wavelength), we observed how the coating materials’ mechanical behavior affected the results.

## 2. Materials and Methods

### 2.1. Coating Material of FBGSs

**Figure 1:**
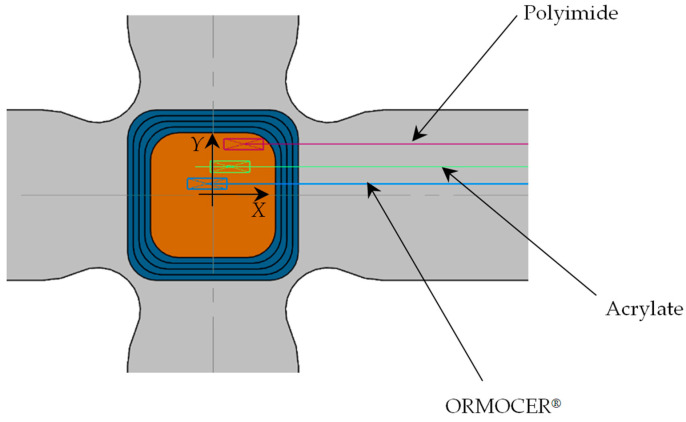
FBG sensors installed on the specimen central zone.

In this study, three FBG sensors with different coatings were installed: polyimide, acrylate, and ORMOCER^®^. Sensors with polyimide and ORMOCER^®^ coatings were manufactured by FBGS (Jena, Germany) and the acrylate-coated sensor was obtained from the School of Aeronautical and Space Engineering of the Polytechnic University of Madrid. These sensors are widely used for monitoring strains and temperatures applied to structures. They are characterized as isotropic and single-mode FBGSs (Table 1). The three sensors were embedded in a cruciform specimen in the plane of symmetry and installed in the central zone (Figure 1 and Table 2). A single exit of the wiring was left through one of the specimen’s arms, which, when placed in the jaws of the triaxial testing machine, ensured a bending radius greater than 30 mm to avoid significant losses during induced light intensity. Table 3 compares the initial values of each sensor measured by our interrogator in the vacuum before being embedded in the specimen and after the curing process in a forced air circulation oven. A variation in wavelengths was observed due to residual stresses originating in the circulation oven curing process, decreasing by ≈500 pm in the polyimide and acrylate sensors and ≈250 pm in ORMOCER^®^.

### 2.2. Experimental Setup

**Figure 2:**
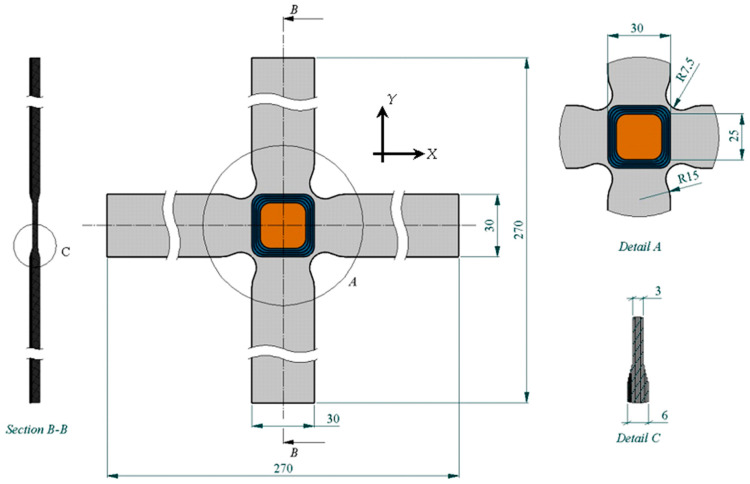
Specimen dimensions and detailed views.

**Figure 3:**
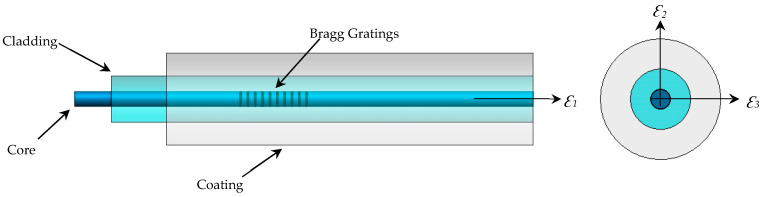
Parts of the Fiber Bragg Gratings Sensors (FBGSs).

The cruciform specimens used in this study were manufactured from CFRPs (Carbon-Fiber-Reinforced Plastics) using unidirectional tape ref. UD UTC–200 and an epoxy resin ref. Ampreg–26 with the slow hardener *Gurit*, whose mechanical properties were experimentally obtained by the Composite Materials Laboratory of the National Institute of Aerospace Technology (INTA), according to ASTM–D3039, ASTM–D3518, ASTM–D2344, and ASTM–D695 [57,58,59,60]. The design of the cruciform specimen was based on previous research [61,62] (see Figure 2 with dimensions in millimeters). The manufacturing or lamination process used was wet, with a lamination sequence of [0°/90°]_10s_. The curing process involved applying a vacuum bag at a pressure of 930 mbar at a temperature of 20 °C for 24 h. After curing, an autoclave post-curing process was conducted at a temperature of 50 °C with a ramp of 3 °C/min, maintaining a temperature of 50 °C ± 5 °C for 16 h, with a glass transition temperature of $T_{g}=$ 73.9 °C. The FBG sensors (with polyimide, acrylate, and ORMOCER^®^ coatings) were installed in the central area of the specimens’ plane of symmetry in the direction of the composite laminate fiber in a non-aligned manner with distances between them, as shown in Figure 1. Finally, a sensor output terminal coinciding with one of the arms of the cruciform specimen was left so that the connectors for the HBM SI405 optical interrogator could be installed. Table 4 shows fiber optics’ physical properties, and Figure 3 shows the parts that comprise an FBGS.

**Figure 4:**
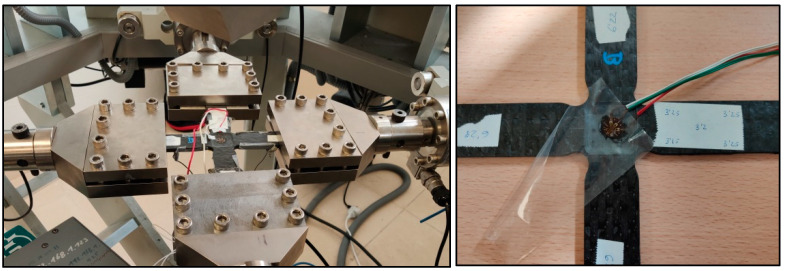
Biaxial test in the horizontal plane of the triaxial machine and a cruciform specimen with a strain gauge rosette and FBGSs.

The testing setup consisted of a Microtest triaxial machine and model EM6/50/FR/SCM for biaxial tests, using four actuators in the horizontal plane (Figure 4). This machine was located in the Testing Laboratory of Continuous Media Mechanics at the University of Castilla—La Mancha. Additionally, a strain gauge rosette was installed on one side and in the central zone of the specimens (Figure 4) to measure the strains. The rosette was connected to an extensometry data acquisition system PCD-300B from KYOWA™ and measured using a microscope from Vision ENGINEERING Ltd. (Woking Surrey, UK) and a digital readout system from Quadra-Check^®^ 200 from METRONICS^®^ (Dublin, Ireland). The deviation from the orientation of the x- and y-axes of the cruciform specimens is approximately 0° 8′. The HBM SI405 optical interrogator has four channels, three of which were used in this study. The output signals obtained from the interrogator during the test were recorded using Micron Optics ENLIGHT version 1.18.8.0, 32-bit with a sample rate of 5 Hz.

### 2.3. Strain–Optic Theory

According to reference [56] and applying the equations to an isotropic sensor, we determined that variations in the average ${(\Delta }^{s}n_{avg})$ and differential ${(\Delta }^{s}n_{diff})$ refractive coefficients of the study grating were as follows:

(1) $$\Delta^{s}n_{avg}=-\frac{n_{0}^{3}}{2}\left(p_{12}\varepsilon_{1}+\left(p_{11}+p_{12}\right)\frac{\varepsilon_{2}+\varepsilon_{3}}{2}\right)$$

(2) $$\Delta^{s}n_{diff}=-n_{0}^{3}\frac{p_{11}-p_{12}}{4}\;\sqrt{{\left(\varepsilon_{2}-\varepsilon_{3}\right)}^{2}+\gamma_{23}^{2}}$$

where $n_{0}$ is the index of the refraction initial; $\varepsilon_{i}$ is the strain field with index 1–3 (index 1 corresponds to the optic fiber direction and indexes 2–3 are oriented perpendicular to the sensor direction); the term $\left(\sqrt{{\left(\varepsilon_{2}-\varepsilon_{3}\right)}^{2}+\gamma_{23}^{2}}\right)$ is the maximum shear strain in the sensor perpendicular to the sensor axis; and $p_{11}$ and $p_{12}$ are the Pockel constants of an isotropic sensor tested at a constant temperature. In the previous equations, variations in the sensor’s refractive coefficients did not consider residual strains, which is typical of the curing of the matrix since they are always present during the tests. Considering a straight Bragg sensor and using the equations of Kim et al. [56], the normalized variation in the mean and differential Bragg wavelength variation in the two components can be expressed as follows:

(3) $$\frac{\Delta^{s}\lambda_{B,avg}}{\lambda_{B0}}=\varepsilon_{1}+\frac{\Delta n_{avg}}{n_{0}}=\left(1-\frac{n_{0}^{2}}{2}p_{12}\right)\varepsilon_{1}-\frac{n_{0}^{2}}{4}(p_{11}+p_{12})(\varepsilon_{2}+\varepsilon_{3})$$

(4) $$\frac{\Delta^{s}\lambda_{B,diff}}{\lambda_{B0}}-n_{0}^{2}\frac{p_{11}-p_{12}}{4}\;\sqrt{{\left(\varepsilon_{2}-\varepsilon_{3}\right)}^{2}+\gamma_{23}^{2}}$$

According to these equations, two effects occur when a strain is in a transverse direction to the fiber, keeping it constant in the longitudinal direction of the fiber. Firstly, there is a displacement of the two components $\vec{p}$ and $\vec{q}$ (displacement vectors perpendicular to the fiber direction), reducing the wavelengths of both. Secondly, splitting occurs in the peaks due to the presence of the transverse forces. The splitting of the peaks should be minimal since there is no cut and the strains in the transverse plane of the fiber are similar. Additionally, the term $n_{0}^{2}\frac{p_{11}-p_{12}}{4}<\left(1-\frac{n_{0}^{2}}{2}p_{12}\right)$.

It is important to note that the strains described in this section are those on the surface of the fiber and not those observed in laminates in general. A strain gauge measures the strains in specimen walls, not the strains felt by the fiber. Therefore, a transformation function is needed to relate the strains in the sheet, where the fiber is located, to the strains experienced by the fiber. Van Steenkiste et al. [63] treated the cross-section of the fiber as if it were an inclusion before applying the equations of Lekhnitskii [64].

## 3. Experimental Results

### 3.1. Testing Plan

**Figure 5:**
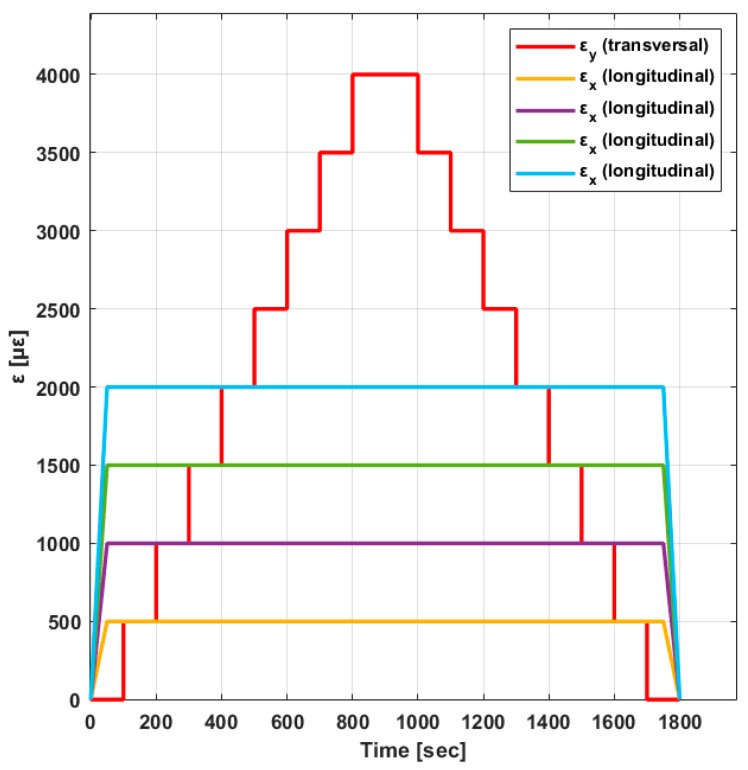
Biaxial design strain values.

These tests analyzed the FBGS’s behavior in a state of biaxial strain. They also analyzed the transverse influence of the output signals on the sensor and the influence of different coatings on the results. For this purpose, the cruciform specimens underwent biaxial tests with a longitudinal strain ($\varepsilon_{x}$) at constant values of 500 με, 1000 με, 1500 με, and 2000 με. For each longitudinal strain state, the transverse strain $\left(\varepsilon_{y}\right)$ varied in steps of 500 με. The transverse strain obtained values from 0 με to 4000 με, maintaining a 2 min break for each step. During the tests, an average temperature of 18.5 °C was recorded with constant humidity values. The loads were applied at a speed of 0.5 $mm\;{min}^{-1}$ (loading and unloading). The test plan consisted of six biaxial tests for each longitudinal strain value (Figure 5).

**Figure 6:**
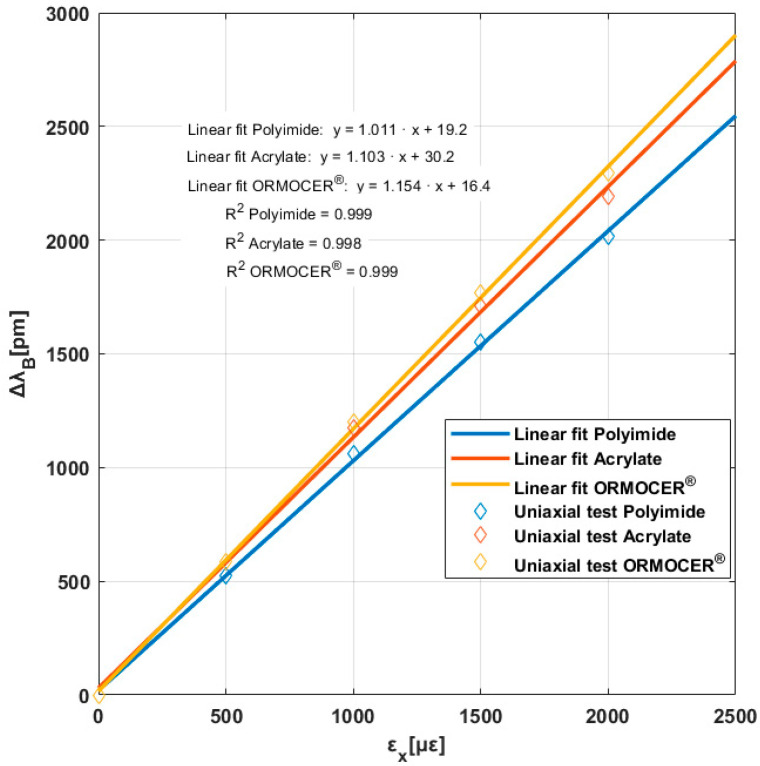
Bragg wavelength increment versus the longitudinal strain for three coatings.

To obtain the K (strain sensitivity factor) of each FBGS, we followed a standard procedure involving a uniaxial test in the direction of FBGSs. In this work, uniaxial tests were performed on the same cruciform specimen, with loading and unloading of up to 2000 με in the arms where the optical fiber is installed, leaving the two arms perpendicular to them free. Figure 6 illustrates our results in a linear interpolation model.

Using the equations in Section 2.3, we found a Bragg wavelength of $\lambda_{B0}=1535\;\mathrm{nm}$, and defining the values of different parameters ($p_{11}=0.113;\;p_{12}=0.252;\;n_{0}=1.449;\;\nu^{s}=0.16$), we determined the following:

(5) $$\Delta \lambda_{B}≃1.2\varepsilon_{1}$$

Table 7 shows the K strain sensitivity factor values for each coating ($K_{Polyimide}=1.011\;pm{\mu \varepsilon }^{-1}$; $K_{Acrylate}=1.103\;pm{\mu \varepsilon }^{-1}\;$and${\;K}_{ORMOCER}®=1.154\;pm{\mu \varepsilon }^{-1}$), which were lower than the theoretical values (1.2 $\;pm{\mu \varepsilon }^{-1}$).

These data are logical since ORMOCER^®^ has greater rigidity than acrylate. Acrylate is higher than polyimide. The differences between the theoretical and experimental slopes are due to two reasons: First, the coating, not being completely rigid, causes a strain gradient between the optical fiber and the material around it. Secondly, the strain measured in the tested specimens was measured using a strain gauge installed on the surface of the specimens’ central area. The strain of the specimen on its surface ${(\varepsilon }^{\infty })$ is different from that experienced by the fiber on its outer surface$\;{(\varepsilon }^{s})$. Therefore, there would be a strain gradient between the fiber coating walls and the specimen strain as well as a strain gradient between the outer walls of the coating and the fiber optic surface (cladding).

In the experimental tests conducted in this study, the longitudinal strain $\varepsilon_{x}$ was constant, while the transverse strain $\varepsilon_{y}$ varied by different values (see Figure 5). The strain field was transmitted to the optical fiber; $\varepsilon_{1}\approx \varepsilon_{x},\;\varepsilon_{2}\approx \varepsilon_{y}\;$and the component in the direction perpendicular $(\varepsilon_{3})\;$to $\varepsilon_{1}\;$and $\varepsilon_{2}$ were affected by Poisson effects. Under these conditions, factor$\;\Delta \lambda_{B}/\lambda_{B}$ for an $\varepsilon_{1}$ constant would involve a decreasing function for an increase in${\;\varepsilon }_{2}$, contrary to the experimental results. It is possible that the transverse strain of the specimens was not transmitted to the FBG sensor, as in the case of strain gauges. Therefore, caution should be exercised when using fiber optics to measure strains in a biaxial strain state.

### 3.2. Biaxial Tests

**Figure 7:**
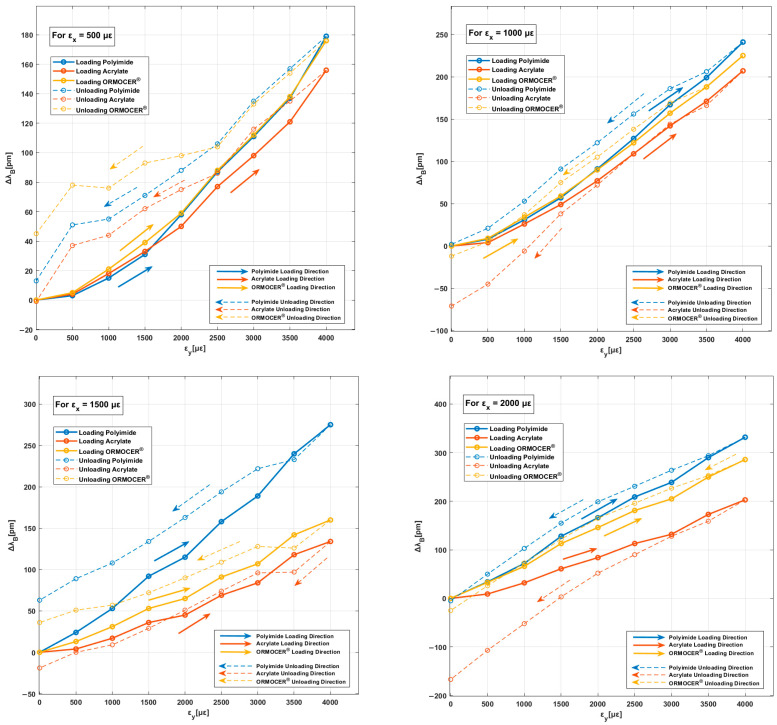
Evolution of $∆\lambda_{B}$ against $\varepsilon_{y}$ for each longitudinal strain state in polyimide, acrylate, and ORMOCER^®^ coatings.

Figure 7 shows the performance of the three sensors installed in each coating for different transverse strain values. According to qualitative analysis, constant values of $\varepsilon_{x}$ (500 με, 1000 με, 1500 με, and 2000 με) for sensor responses ($∆\lambda_{B}$) were not constant as the output values increased. This phenomenon confirms the influence and dependence of the transverse strain $\left(\varepsilon_{y}\right)$ response. The curves would be horizontal if the FBG sensor only had longitudinal strain$\;(\varepsilon_{x})$. With respect to our first study [36], we also observed the coating type’s dependence on sensor response, as indicated by differences in wavelength variation values ($∆\lambda_{B}$) and their response in the download phase. In addition, as in our first study [36], when the longitudinal strain$\;(\varepsilon_{x})$ increased from 500 με to 2000 με, the wavelength variation $(∆\lambda_{B})\;$increased proportionately. In all graphs, the strain states indicate a delay between the loading and unloading curves. This phenomenon may be associated with the mechanical properties of the coating material (Table 1), known as denominated hysteresis. Figure 7 shows the wavelength increment obtained from the initial point of the curves for zero transverse strain.

Table 5 shows the average dependence values of the sensor’s output signals ($∆\lambda_{B}$) on the accumulated transverse strains for longitudinal strain states (loading and unloading). For tests with longitudinal strain values of 500 με, the influence is high with values around 46% for the polyimide coating and 30% for the acrylate and ORMOCER^®^ coatings. A significant difference was noted between values of 500 με in the longitudinal direction and 4000 με in the transverse direction (extreme case). The influence is attenuated in tests with values of 1000 με in the longitudinal direction, with values of around 20%. The results suggest that high transverse–longitudinal strain states significantly impact FBGS behavior and measurements.

The influence of the transverse strain on the FBG sensor behavior can be quantified. Table 6 shows the longitudinal strain obtained in FBGSs for each strain state. It also compares the increase in the Bragg wavelength measured by the interrogator using a calibrated strain sensitivity factor to the strain obtained. For a transverse to longitudinal strain ratio of eight, the estimated error rate for the longitudinal strain is around 56% for the polyimide coating and 30% for acrylate and ORMOCER^®^.

**Figure 8:**
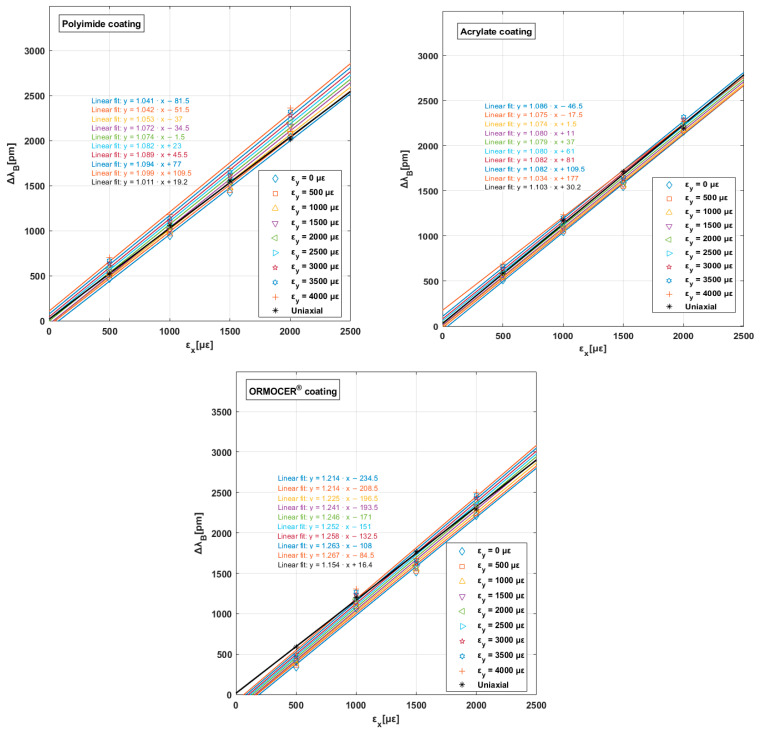
Average values and linear approximation lines with their slopes obtained through biaxial tests for the three coatings.

In Figure 8, the increase in the Bragg wavelength (considering the initial Bragg wavelength as a reference) is shown for the longitudinal strain of each test performed and coating type.

The graph above displays a linear fit for each transverse strain $\left(\varepsilon_{y}\right)$ value up to $\varepsilon_{y}\;$= 4000 με. Furthermore, the ordinate at the origin provided by the linear fit differs for each transverse strain, which demonstrates the important impact of this general strain state on the sensor response. Table 7 lists the values for each coating type. A negative evolution in % K (strain sensitivity factor) was observed in acrylate coatings compared to the other polymers. This value may be justified by its mechanical behavior (see Table 1).

## 4. Conclusions

This study aimed to evaluate the effect of transverse strains on FBG sensor responses embedded in a cruciform specimen of composite material. The analyzed sensors had different coatings (polyimide, acrylate, and ORMOCER^®^) commonly used in structural monitoring. The characterization of this sensor type is usually conducted with standard uniaxial stress tests. This type of test allows the relationship between the longitudinal strain in the sensor to be obtained $\varepsilon_{x}$ and its physical response $∆\lambda_{B}$, which is defined through K (strain sensitivity factor) by applying linear regression to the experimentally obtained points. This value depends on the sensor coating material and is considered constant for the entire strain field.

In uniaxial tests, the sensor is subjected to the transverse strain $\varepsilon_{y},$ which depends on the longitudinal strain $\varepsilon_{x},$ the Poisson coefficient μ, and that it will be equal to${\;\varepsilon }_{y}=-\varepsilon_{x}\mu$. This transverse strain has the following characteristics:

- It presents a fixed value for each longitudinal strain value${\;\varepsilon }_{y}=-\varepsilon_{x}\mu$;
- It presents negative values for each longitudinal strain value μ>0;
- It has low proportions$\;\frac{\varepsilon_{y}}{\varepsilon_{x}}$.

The above characteristics do not correspond to situations found in real structures where they are common due to complex load states, having different transverse strain values for the same longitudinal strain value, or situations in which the transverse strain may be higher than the longitudinal strain. In laminate composite structures with thin thicknesses, we can assume the plane stress hypothesis when subjected to loads contained in the plane. For this reason, a scientific methodology was conducted to develop different plane stress cases using biaxial tests. We reached the following conclusions from our results:

- The response of the sensor $∆\lambda_{B}$ to the longitudinal $\varepsilon_{x}$ strain was significantly influenced by the transverse strain $\varepsilon_{y}$ and the coating material. The influence of the transverse strain affected three fundamental parameters of the sensor: the output or response of the sensor $∆\lambda_{B}$ and two derived values, such as the sensor’s K (strain sensitivity factor) and the interpreted με value. The influence of the transverse strain on the sensor’s response $∆\lambda_{B}$ can reach values of up to 46% in the signal for the defined reference state (the one with a ratio $\frac{\varepsilon_{y}}{\varepsilon_{x}}=0$). This extreme case was observed in a polyimide-coated sensor subjected to a ratio of $\frac{\varepsilon_{y}}{\varepsilon_{x}}=8$. At lower ratios, the influence decreases. We also observed that the influence on the sensor’s output signal was lower in acrylate and ORMOCER^®^ coatings, exhibiting similar behaviors at around 30%.
- Regarding magnitudes derived from sensor K (strain sensitivity factor) and με interpretation, the influence can reach a 10% increase in extreme cases $\left(\varepsilon_{y}=4000\;\mu \varepsilon \right)$ for polyimide and ORMOCER^®^ coatings. On the other hand, for the acrylate coating, a decrease of 6% in the sensor’s K value (strain sensitivity factor) was observed. This phenomenon may be due to the mechanical nature of the coating material (Table 1).
- A significant hysteresis effect was observed in the loading and unloading cycles of the acrylate coating, being higher than 150 pm in one case, which is logical considering the less rigid nature (Table 1) of this polymer.
- Based on the results obtained, the standardized sensor characterization procedure should be reconsidered for sensors working in multiaxial stress states with high $\frac{\varepsilon_{y}}{\varepsilon_{x}}$ ratios, where the sensor’s K (strain sensitivity factor) could lead to erroneous interpretations in terms of interpreted με.

When compared to the Strain–Optic Theory, these experimental results demonstrated that the increase in the Bragg wavelength recorded followed a trend contrary to the theory equations. One possible cause may be that the strain field was not completely transferred to the FBGS in traction but in out-of-plane compression. To analyze and study this phenomenon, as well as the sensor’s response, different studies involving states of deformation applied to compression or an equivalent are necessary to confirm or discard this hypothesis.

## Figures and Tables

**Table 1:** Physical properties of the polyimide, acrylate, and ORMOCER^®^ coatings used in this study [1,39,40].

Properties	Units	Polyimide	Acrylate	ORMOCER^®^
Young’s modulus (E)	GPa	2.40	0.60	2.00
Density	$g/{cm}^{3}$	1.43	[1.14–1.20]	Not available
Temperature glass transition	°C	>400	≈105	250
Temperature of fusion	°C	Not available	[160–200]	Not available
Vicat softening temperature (VST)	°C	220	Not available	Not available
Operative range of temperature	°C	[−190–350]	[−55–85]	[−180–200]
Core diameter	µm	9	9	6
Cladding diameter	µm	125	125	125
Coating diameter	µm	160	250	200

**Table 2:** Absolute coordinates since zero reference (mm).

Coatings	X	Y
Polyimide	5.4	9
Acrylate	3	5
ORMOCER^®^	−1	2

**Table 3:** Values of $\lambda_{B}$ for FBGSs with the polyimide, acrylate, and ORMOCER^®^ coatings used in this study.

Properties	Units	Polyimide	Acrylate	ORMOCER^®^
Pre-installation	nm	1535.004	1562.028	1546.816
Post-installation	nm	1534.624	1561.634	1546.578

**Table 4:** FBGS properties used in this study [1,36].

Properties	Units	Values
$\mathrm{Young}’s\;\mathrm{modulus}\;(E^{s}$)	GPa	73.1
$\mathrm{Poisson}’s\;\mathrm{ratio}\;(\nu^{s}$)		0.16
$\mathrm{Shear}\;\mathrm{modulus}\;(G^{s}$)	GPa	31.5
$\mathrm{Thermal}\;\mathrm{expansion}\;\mathrm{coefficient}\;(\alpha^{s}$)	$10^{-6}/{}^{\circ}C$	0.5
$\mathrm{Index}\;\mathrm{of}\;\mathrm{refraction}\;(n_{0}$)		1.449
$\mathrm{Pockel}\;\mathrm{constant}\;(p_{11}$)		0.113
$\mathrm{Pockel}\;\mathrm{constant}\;(p_{12}$)		0.252
$\mathrm{Thermo}-\mathrm{optic}\;\mathrm{coefficient}\;(\frac{dn_{0}}{dT}$)	$10^{-5}/{}^{\circ}C$	0.83

**Table 5:** Average percentages of accumulated transverse strains for each longitudinal strain tested.

**Polyimide**	**Loading**				**Unloading**			
	**500 [με]**	**1000 [με]**	**1500 [με]**	**2000 [με]**	**2000 [με]**	**1500 [με]**	**1000 [με]**	**500 [με]**
	46.4%	23.1%	17.5%	15.8%	14.9%	13.7%	22.4%	42.5%
**Acrylate**	**Loading**				**Unloading**			
	**500 [με]**	**1000 [με]**	**1500 [με]**	**2000 [με]**	**2000 [με]**	**1500 [με]**	**1000 [με]**	**500 [με]**
	33.3%	17.8%	8.1%	8.8%	16.3%	9.9%	25.2%	29.4%
**ORMOCER** ^®^	**Loading**				**Unloading**			
	**500 [με]**	**1000 [με]**	**1500 [με]**	**2000 [με]**	**2000 [με]**	**1500 [με]**	**1000 [με]**	**500 [με]**
	30.6%	19.9%	9.9%	12.4%	12.9%	7.8%	21.4%	26.1%

**Table 6:** Experimental values measured by the interrogator with different ratios and coatings.

**Polyimide**	**Reference Values**		**Ratio**	**Strain in the Specimen**	**Bragg Wavelength Increment**	**Strain from FBGS**	**Estimation Error**
	$\varepsilon_{x}\left[\mu \varepsilon \right]$	$\varepsilon_{y}\left[\mu \varepsilon \right]$	$\varepsilon_{y}/\varepsilon_{x}$	$\varepsilon_{x}\left[\mu \varepsilon \right]$	$∆\lambda_{B}\left[pm\right]$	$\varepsilon_{x}\left[\mu \varepsilon \right]$	**%**
	500	4000	8	500	792.6	784	56.7
	500	3000	6	500	739	731	46.3
	1000	4000	4	1000	1251.6	1238	23.8
	1000	2000	2	1000	1101.9	1090	9.0
	1000	1000	1	1000	1042.3	1031	3.1
**Acrylate**	Reference values		Ratio	Strain in the specimen	Bragg wavelength increment	Strain from FBGS	Estimation error
	$\varepsilon_{x}\left[\mu \varepsilon \right]$	$\varepsilon_{y}\left[\mu \varepsilon \right]$	$\varepsilon_{y}/\varepsilon_{x}$	$\varepsilon_{x}\left[\mu \varepsilon \right]$	$∆\lambda_{B}\left[pm\right]$	$\varepsilon_{x}\left[\mu \varepsilon \right]$	**%**
	500	4000	8	500	707	641	28.3
	500	3000	6	500	662.9	601	20.1
	1000	4000	4	1000	1299.3	1178	17.8
	1000	2000	2	1000	1175.8	1066	6.6
	1000	1000	1	1000	1122.8	1018	1.8
**ORMOCER^®^**	Reference values		Ratio	Strain in the specimen	Bragg wavelength increment	Strain from FBGS	Estimation error
	$\varepsilon_{x}\left[\mu \varepsilon \right]$	$\varepsilon_{y}\left[\mu \varepsilon \right]$	$\varepsilon_{y}/\varepsilon_{x}$	$\varepsilon_{x}\left[\mu \varepsilon \right]$	$∆\lambda_{B}\left[pm\right]$	$\varepsilon_{x}\left[\mu \varepsilon \right]$	**%**
	500	4000	8	500	564.9	652	30.4
	500	3000	6	500	688.9	597	19.4
	1000	4000	4	1000	1321.3	1145	14.5
	1000	2000	2	1000	1244	1078	7.8
	1000	1000	1	1000	1187.5	1029	2.9

**Table 7:** K values (strain sensitivity factor) for each fixed transverse strain value and evolution percentage.

**Polyimide**	**Transverse strain**	**Uniaxial**	**0**	**500**	**1000**	**1500**	**2000**	**2500**	**3000**	**3500**	**4000**	**% *K***
	***ε_y_* [με]**											
	Slope experimental results	1.011	1.041	1.042	1.053	1.072	1.074	1.082	1.089	1.094	1.099	8.70
	**[pm με^−1^]**											
	Ordinate in the origin	19.2	−81.5	−51.5	−37	−34.5	−1.5	23	45.5	77	109.5	-
	**[pm]**											
**Acrylate**	Transverse strain	Uniaxial	0	500	1000	1500	2000	2500	3000	3500	4000	% *K*
	***ε_y_* [με]**											
	Slope experimental results	1.103	1.086	1.075	1.074	1.080	1.079	1.080	1.082	1.082	1.037	−5.98
	**[pm με^−1^]**											
	Ordinate in the origin	30.2	−46.5	−17.5	1.5	11	37	61	81	109.5	177	-
	**[pm]**											
**ORMOCER^®^**	Transverse strain	Uniaxial	0	500	1000	1500	2000	2500	3000	3500	4000	% *K*
	***ε_y_* [με]**											
	Slope experimental results	1.154	1.214	1.214	1.225	1.241	1.246	1.252	1.258	1.263	1.267	9.79
	**[pm με^−1^]**											
	Ordinate in the origin	16.4	−234.5	−208.5	−196.5	−193.5	−171	−151	−132.5	−108	−84.5	-
	**[pm]**											

## Data Availability

Data are contained within the article.
